# *N*-acetylcysteine: a novel approach to methaemoglobinaemia in normothermic liver machine perfusion

**DOI:** 10.1038/s41598-023-45206-z

**Published:** 2023-11-03

**Authors:** George Clarke, Jingwen Mao, Yiyu Fan, Angus Hann, Amita Gupta, Anisa Nutu, Erwin Buckel, Kayani Kayani, Nicholas Murphy, Mansoor N. Bangash, Anna L. Casey, Isla Wootton, Alexander J. Lawson, Bobby V. M. Dasari, M. Thamara P. R. Perera, Hynek Mergental, Simon C. Afford

**Affiliations:** 1 Liver Unit, Queen Elizabeth Hospital Birmingham, Mindelsohn Way, Birmingham, B15 2TH UK; 2 Birmingham Biomedical Research Centre, National Institute for Health Research (NIHR), University of Birmingham and University Hospitals Birmingham NHS Foundation Trust, Birmingham, B15 2TH UK; 3 Centre for Liver and Gastrointestinal Research, Institute of Immunology and Immunotherapy, University of Birmingham, Birmingham, B15 2TH UK; 4 Ochre-Bio Ltd, Oxford, UK; 5 Queen Elizabeth Hospital Birmingham, Birmingham, B15 2TH UK; 6 Intensive Care Unit, Queen Elizabeth Hospital Birmingham, Birmingham, B15 2TH UK; 7 Birmingham Acute Care Research Group, Institute of Inflammation and Ageing, University of Birmingham, Birmingham, B15 2TH UK; 8 Microbiology Department, Queen Elizabeth Hospital Birmingham, Birmingham, B15 2TH UK; 9 Clinical Biochemistry, Queen Elizabeth Hospital Birmingham, Birmingham, B15 2TH UK

**Keywords:** Liver, Preclinical research, Physiology

## Abstract

Extended duration of normothermic machine perfusion (NMP) provides opportunities to resuscitate suboptimal donor livers. This intervention requires adequate oxygen delivery typically provided by a blood-based perfusion solution. Methaemoglobin (MetHb) results from the oxidation of iron within haemoglobin and represents a serious problem in perfusions lasting > 24 h. We explored the effects of anti-oxidant, *N*-acetylcysteine (NAC) on the accumulation of methaemoglobin. NMP was performed on nine human donor livers declined for transplantation: three were perfused without NAC (no-NAC group), and six organs perfused with an initial NAC bolus, followed by continuous infusion (NAC group), with hourly methaemoglobin perfusate measurements. In-vitro experiments examined the impact of NAC (3 mg) on red cells (30 ml) in the absence of liver tissue. The no-NAC group sustained perfusions for an average of 96 (range 87–102) h, universally developing methaemoglobinaemia (≥ 2%) observed after an average of 45 h, with subsequent steep rise. The NAC group was perfused for an average of 148 (range 90–184) h. Only 2 livers developed methaemoglobinaemia (peak MetHb of 6%), with an average onset of 116.5 h. Addition of NAC efficiently limits formation and accumulation of methaemoglobin during NMP, and allows the significant extension of perfusion duration.

## Introduction

The disparity between the rising need for life-saving liver transplantation and the limited availability of quality organs are increasingly met by the use of extended criteria donors (ECDs). Dynamic liver preservation using machine perfusion has been shown to provide benefit to these suboptimal donor organs. Normothermic machine perfusion (NMP) has enabled both the functional testing of organs and the extension of preservation time prior to transplantation^1–3^.

The use of NMP as a logistical tool is increasing. Organs are being perfused over longer periods of time, in many cases approaching 24 h, with one published case reporting successful transplantation following 68 h of NMP^4^. Whilst there is currently a limited need to extend liver preservation times beyond 24 h in clinical practice, this is likely to become a new frontier given that it may allow liver bioengineering, biobanking, and on-demand access to organs in the future.

Several teams have already embarked on research into extended liver preservation (defined as a perfusion time in excess of 24 h), successfully achieving perfusions beyond 5 days^5,6^. Extending the perfusion time requires substantial amendments to the NMP circuit, revised perfusion protocols, liver monitoring, and perfusate parameter adjustments that far exceeds that used in the currently established clinical perfusions^5,7–9^.

**Figure 1:**
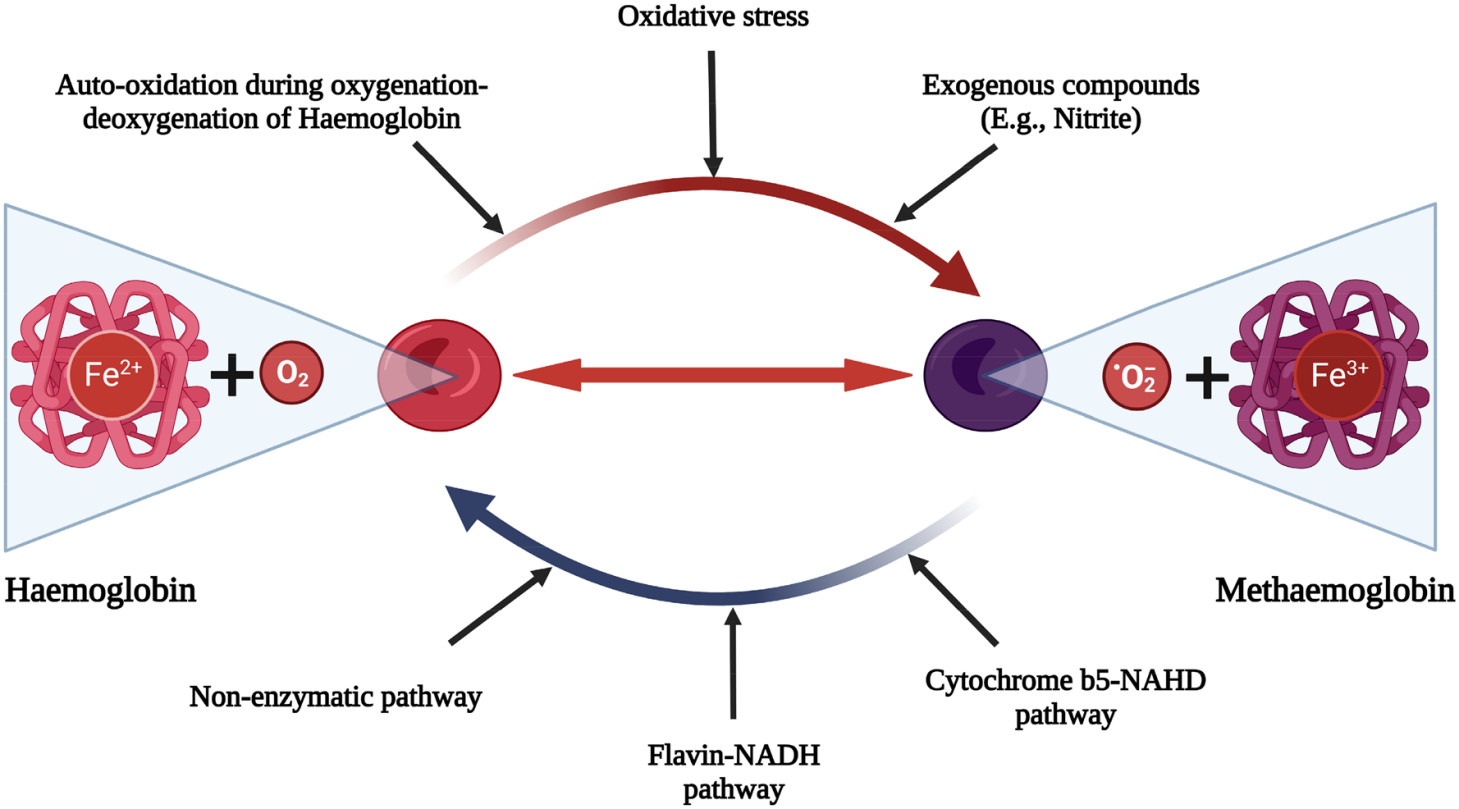
Conversion between haemoglobin and methaemoglobin. Methaemoglobin is oxidised haemoglobin with the iron in Fe^3+^ form, and it is incapable of carrying oxygen. In normal circumstance, methaemoglobin is produced spontaneously in red blood cells and can be reduced back to haemoglobin through enzymatic pathway. The level of methaemoglobin is tightly regulated by an intricate balance of mechanisms that produce and reduce methaemoglobin. Figure created using BioRender.

Developing new perfusion protocols becomes a dynamic process aimed at overcoming the numerous unexpected barriers that only present themselves during long-term perfusion experiments. One such complication is the insidious development of methaemoglobinaemia. Methaemoglobinaemia (methaemoglobin [MetHb] levels in excess of 2%) represents a rare haematological disorder in humans due to the oxidation of haemoglobin (Hb)^10^. The most common cause of methaemoglobinaemia is exposure to an oxidising agent, such as in nitrite poisoning, however it can also be caused by a variety of genetic defects affecting both red cell metabolism and Hb structure^3^. In its normal state, tetrameric Hb- consists of four haem-globin complexes, each haem containing a single iron atom is in its reduced ferrous state (Fe^2+^). However, once oxidized to a ferric state (Fe^3+^) the Hb subunit’s oxygen-binding ability is lost (Fig. 1) while the remaining 3 subunits in the Hb tetramer retain bound O_2_ more avidly, further diminishing its delivery to tissues. MetHb is not a permanent state and red cells are able to utilise NADH methaemoglobin reductase to reduce iron back into its ferrous state. Accumulation of MetHb and methaemoglobinaemia occurs once this protective system fails.

In the context of extended NMP the formation of methaemoglobinaemia has already been reported^11^. Its presence is especially notable in suboptimal or marginal organs and its insidious production can lead to impaired oxygen delivery and possible failure. Methylene blue, first line treatment in a clinical situation, has been demonstrated ineffective in reducing perfusion-associated methaemoglobinaemia and currently there is no established intervention to address this problem during NMP^11^.

Upon developing our extended NMP protocol, our initial three perfusions failed in association with high levels of MetHb. We hypothesised that the formation of MetHb may be prevented by the addition of a compound with anti-oxidative properties to the NMP perfusate. *N*-acetylcysteine (NAC) has potent anti-oxidant properties and is used in the management of paracetamol-induced liver injury^12^. It neutralizes free oxygen radicals and replenishes cellular stores of glutathione^13,14^. NAC has been experimented with during cold preservation of donor livers due to its anti-oxidant effects, however no significant benefits was observed in prospective studies^15^.

To test our hypothesis, we designed 5-days NMP liver perfusion experiments using discarded donor livers with addition of NAC and sequential measurements of MetHb levels. In addition, we tested the effect of NAC on MetHb formation during in-vitro experiments using packed red cells.

## Methods

### Donor livers and research ethical approval

All packed red cells and donor livers were sourced from the National Health Service Blood and Transplant (NHSBT). Discarded donor livers were initially retrieved with the intention of transplantation as per NHSBT guidelines. Following decline for transplantation by all UK centres the organs were offered nationally for research by NHSBT. Informed consent for use in research was obtained from donor families by the specialist nurse in organ donation in accordance with NHSBT guidelines, and this study was approved by the London-Surrey Borders National Research Ethics Service (reference 18/WA/0214) and the NHSBT ethics committee (reference 06/Q702/61). All methods were performed in accordance with the relevant guidelines and regulations at the University of Birmingham. All organs were preserved and transported in University of Wisconsin preservation solution at 4 °C.

### *N*-acetylcysteine and packed red cells in-vitro (without liver tissue)

**Figure 2:**
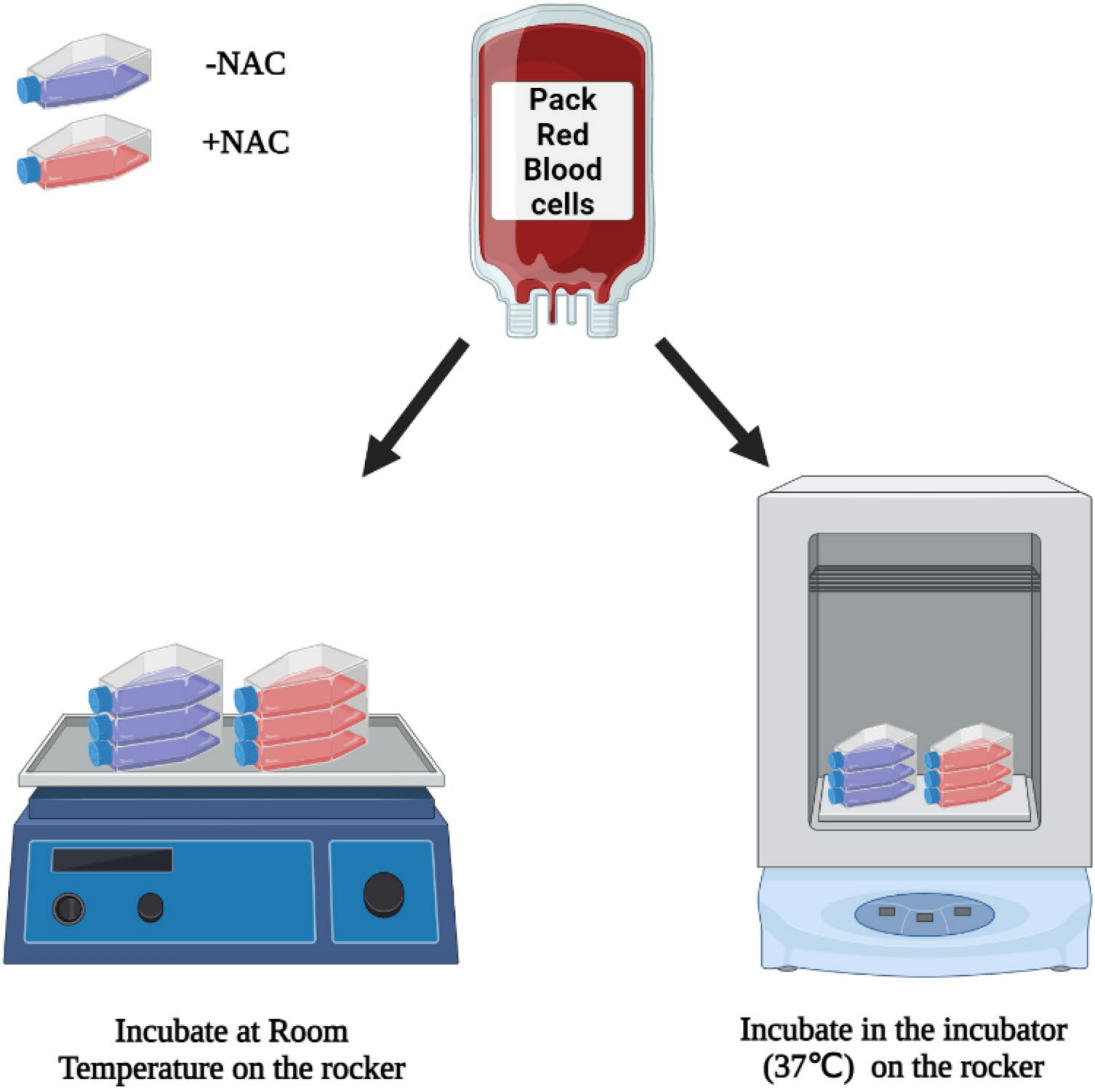
In vitro experiment. Thirty ml O-negative blood were added to 12 T25 flasks, and initial MetHb level is measured. NAC (3 mg) is added to 6 of the flasks. These 6 flasks with or without NAC then sub-divided into 2 groups, one group incubated in the incubator (36 °C) and one group incubated at room temperature (20 °C), all flasks are placed on the horizontal shaker during incubation. MetHb level is measured every hour using a COBAS 221b blood gas analyser. Figure created using BioRender.

An in-vitro experiment was designed to evaluate the effects of NAC on MetHb accumulation in packed red cells in the absence of liver tissue. Thirty millilitres of research grade O negative (O^−^) packed red cells were placed in T25 flasks. Twelve samples were divided into two test groups (A and B): Group A without NAC (*n* = 6) and Group B with NAC (*n* = *6*). Half of each sample group (*n* = *3*) were placed in an incubator at 37 °C, with the remaining kept at room temperature (20 °C) (Fig. 2). Group B had 3 mg of NAC (Parvolex, Meath, Ireland) added to their volume at time 0. Methaemoglobin was measured by hourly blood gas measurements over 6 h using a Cobas b 221 (Roche Diagnostics, Indianapolis, IN) point-of-care system blood gas analyser. Oxygen exchange by packed red cells was reliant on air equilibrium.

### Normothermic machine perfusion

Discarded donor livers were subjected to NMP following variable periods of static cold storage using the Liver Assist device (XVIVO, Gothenburg, Sweden). A custom-made, single-use perfusion kit was used, with dual oxygenators licensed for 14 days. The livers were prepared following standard bench preparation practice and perfusion set-up^16^. The cystic duct was ligated, and an appropriately sized T-tube inserted into the common hepatic duct. The initial perfusate was composed of research-grade O^–^ packed red cells, 5% human albumin solution, and additional drugs as specified in Supplementary Table 1. Livers 4–9 underwent continuous veno-venous haemofiltration during perfusion (Aquarius, Nikkiso, North America, San Diego, CA) to correct electrolyte imbalances and acidosis observed during extended NMP. The intended duration for all perfusions was 5 days.

### Perfusate composition and methaemoglobin assessment

Perfusate MetHb levels were measured using a point-of-care system blood gas analyser in hourly intervals. The initial 3 perfusions (Livers 1–3) were performed without NAC infusion. During those we observed significant MetHb accumulation impacting on liver oxygen delivery and function. Livers 4–9 received a bolus of NAC (200 mg) at the start of perfusion followed by a continuous NAC infusion at a standard rate of 200 mg/hour via a port on the hepatic artery circuit.

### Statistical analysis

All data represent the values of at least three independent experiments. Data are expressed as median ± range and were analysed using GraphPad Prism 9 software. Differences between groups were analysed using the analysis of unpaired T test and Mann Whitney test. A *p* value < 0.05 was considered significant.

## Results

### NAC and packed red cell ex-vivo model

**Figure 3:**
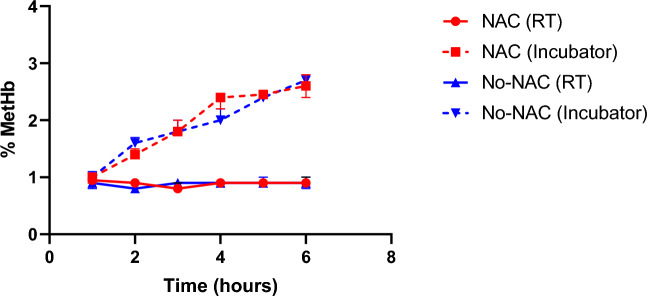
Graph showing MetHb levels during the in-vitro experiments using packed red cells. Comparison is shown between samples incubated at room temperature (RT, 20 °C) and in an incubator (36 °C).

Packed red cells had a starting mean MetHb accumulation of 0.96% and 0.93% in the NAC and no-NAC cohort, respectively. In both groups the lowest MetHb value was at hour 1 and the highest at hour 6. The MetHb levels did not change over the course of the 6 h incubation at room temperature in either NAC or no-NAC cohorts (*p* = 0.317). In the cohort incubated at 37 °C the MetHb levels gradually increased, reaching the highest values, 2.60% and 2.73%, respectively, at 6-h. Whilst there was significant difference between the cumulation based on the temperature, *p* = 0.011 and *p* = 0.006, respectively, there was no difference based on addition of the NAC to the incubation media. The details are provided in Table 1 and Fig. 3.

**Table 1:** Table showing median methaemoglobin values (%) during the in-vitro experiment.

Time (Hour)	Room temp (%)	Incubator (%)	
With *N*-acetylcysteine			
1	0.95	0.97	
2	0.90	1.43	
3	0.83	1.87	
4	0.90	2.33	
5	0.90	2.45	
6	0.93	2.6	*p* = 0.011
Without *N*-acetylcysteine			
1	0.85	1.00	
2	0.80	1.63	
3	0.87	1.87	
4	0.90	2.07	
5	0.93	2.43	
6	0.90	2.73	*p* = 0.006
	*p* = 0.317	*p* = 0.841

### Donor Liver Characteristics and the initial 24-h perfusion details

The livers enrolled in this study consisted of 6 organs donated after brainstem death (DBD) (67%) and 3 organs donated after circulatory death (DCD) (33%). The median static cold storage time before starting NMP was 638 (range 540–867) min. The DCD organs had a mean donor warm ischaemia time of 20 (range 12–34) min.

The median donor age was 60 (range 42–77) years old. Median liver weight was 1.86 (range 1.02–3.57) kg. Details of the donor demographics and reasons for discard are provided in Table 2. Eight out of nine (89%) livers achieved viability as defined by the Birmingham viability criteria^1,17,18^. Liver 6 was observed to have poor hepatic artery flows for the majority of the first 24 h and cleared its lactate below 2 mmol/L by hour 7.

**Table 2:** Donor demographics and details of each donor liver used.

	1	2	3	4	5	6	7	8	9
Donor Type	DBD	DBD	DCD	DBD	DBD	DCD	DBD	DBD	DCD
Sex	Female	Male	Female	Male	Female	Male	Male	Female	Male
Age	52 years	59 years	77 years	61 years	70 years	42 years	73 years	60 years	51 years
Steatosis	Mild–moderate	Mild–moderate	Nil	Moderate	Moderate	Nil	Mild	Moderate	Severe
Liver Weight	1.732 kg	1.936 kg	1.245 kg	1.900 kg	1.024 kg	2.515 kg	1.864 kg	1.688 kg	3.568 kg
Cold Ischaemic Time	638 min	659 min	540 min	680 min	867 min	626 min	622 min	750 min	586 min
Warm Ischaemia Time	–	–	12 min	–	–	34 min	–	–	15 min

### Liver perfusion without *N*-acetylcysteine (livers 1–3)

The initial three perfusions (33% of this series) represent our team’s first attempts at extended NMP, with the median achieved perfusion time being 100 (range 87–102) h. All 3 livers (100%) in the non-NAC group suffered from excessive MetHb accumulation reaching 35–43%. The median time to develop methaemoglobinaemia (> 2%) was 34 (range 31–70) h, with subsequent steep rise.

### Liver perfusion with *N*-acetylcysteine (livers 4–9)

Six livers (67% of this series) were perfused with a continuous infusion of NAC (200 mg/hour). The NAC group had a median peak MetHb accumulation of 1.3%. Only 2 (33%) of NAC infused livers developed methaemoglobinaemia (> 2%)*,* of which the mean time to develop was 116.5 h.

Liver 4 represents an outlier amongst the NAC cohort, with a peak MetHb accumulation of 28%. This liver had a controlled MetHb < 2% until hour 85, with rapid MetHb accumulation until perfusion termination at hour 90 (MetHb 28%). This was associated with a rise in lactate (starting hour 66), and alanine transaminase (ALT, starting hour 66) reflecting organ deterioration and eventual failure.

### Comparison of the NAC and non-NAC groups

**Figure 4:**
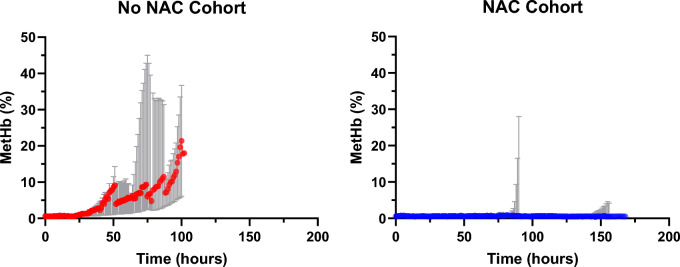
MetHb level during NMP. The figures shows the median and range of both the no NAC cohort (red) and NAC cohort (blue) over 168 h of NMP time. In both figures, the median is shown by the coloured line and the range is displayed in grey.

In the first 12 h there was no significant difference between the NAC cohort and the control cohort (*p* = 0.909), however there was a significant difference between both groups over 24 h, 48 h, 72 h, and 84 h (*p* = 0.0002, *p* < 0.0001, *p* < 0.0001, *p* < 0.0001, respectively) (Fig. 4).

## Discussion

This is the first series reporting the protective impact of NAC on the accumulation of methaemoglobin in extended NMP of donor livers. Severe methaemoglobinaemia is a progressive complication of extended NMP. This has previously been reported in shorter clinical perfusions, most notably in suboptimal donors, and is thought to be a universal complication during NMP^11^. We encountered this problem during our development of a 5-days NMP model, where initial perfusions demonstrated universal accumulation of methaemoglobin beyond 48-h perfusions, with subsequent sharp rise in levels.

MetHb build up in our series was also accompanied with a progressive increase in carboxyhaemoglobin leading to a gradual decline in the oxyhaemoglobin (O_2_Hb) and the impairment of essential oxygen delivery. Certainly, if left untreated, this gradual decline in oxyhaemoglobin would eventually lead to tissue hypoxia, graft injury, and potential failure. Livers 1–4 were stopped due to features of progressive organ failure (severe lactic acidosis, increasing vascular resistance, and parenchymal changes), whereas Livers 5–9 were stopped following meeting our perfusion time targets (initially 120 h, extended to 144 h and 168 h). The reasons for liver failure during machine perfusion has yet been unexplored and multifactorial with methaemoglobinaemia and poor oxygen delivery playing a role.

Interestingly, NAC was a component of the perfusion protocol in Tingle et al.^11^ report of severe methaemoglobinaemia, and did not prevent its development. Presumably, the authors used a single bolus of acetylcysteine into the perfusate reservoir when commencing the perfusion. NAC has three main mechanisms of action: as a free radical scavenger; a precursor for glutathione biosynthesis; and a reducer of disulfide bonds^14^. A combination of both NAC’s anti-oxidant properties and replenishment of glutathione is hypothesised to subdue the MetHb accumulation seen.

It has been hypothesised that NAC does not require conversion to glutathione to exert its effects on MetHb^19^. This is not supported entirely by in-vitro studies, with evidence both for and against its effectiveness^20,21^. The results in our study are mixed, with our in-vitro experiment highlighting the need for viable hepatocytes to facilitate the anti-oxidant benefit of NAC. We observed that even in the presence of NAC there was production of MetHb in line with packed red cells without treatment. When replicating our in-vitro study with glutathione (3 mg in 30 ml of research grade O^–^ packed red cells), we did not see significant difference in MetHb accumulation between no-glutathione and glutathione groups (data not included). Although unclear and undefined at present, the mechanism behind NAC’s effectiveness in suppressing MetHb accumulation is not solely due to its conversion to glutathione and requires the presence of healthy hepatocytes. Methaemoglobinaemia does not appear to be a relevant problem in the initial 24 h of NMP and we hypothesise that NAC supports an intrinsic protective pathway present within healthy hepatocytes.

NAC undergoes extensive first pass metabolism in the liver and kidneys leading to low levels in the circulating plasma^22,23^. This supports our hypothesis that a continuous infusion rather than one-off bolus may be the preferred way to supplement NAC during extended NMP, even in the absence of continuous veno-venous haemofiltration. It has a low volume and there should be no risk of excessive dilution of the circuit.

This study has some limitations to consider when interpreting the results. One shortcoming is the evolution of the perfusion protocol alongside our learning curve. Following the landmark report by the Zurich group we added continuous veno-venous haemofiltration and methylprednisolone to our protocol (Supporting Table 1). This appeared to have an important role in increasing the success of the extended NMPs and to some extent this overlaps with the addition of NAC to our perfusion protocol.

Given the multiple constraints, including the access to discarded donor livers, financial resources, time, and labour intensity, it is not feasible in the real-world to perform a study to demonstrate the benefits of each specific component of the perfusion fluid. Other potentially usable anti-oxidant additives might be vitamin C, vitamin K, lipoic acid, and glutathione, having the ability to neutralise oxygen free radicals, however NAC has the additional benefit of cysteine replenishment allowing the manufacture of glutathione^24^.

Although outside the scope of this manuscript, the benefits of NAC are likely to exceed suppression of methaemoglobin accumulation alone. This safe, cheap, and efficient drug has previously been used in some fluids developed for cold organ preservation to reduce ischaemia reperfusion injury. A major component of this process is the production of reactive oxygen species that aggravates tissue damage^25^. NAC is likely to attenuate the pro-oxidant environment and damage cascade initiated during liver reperfusion, which might be of further benefit whilst using the NMP in extended criteria donors.

## Conclusion

Methaemoglobinaemia is a universal complication of extended NMP with all organs developing severe methaemoglobinaemia in the absence of a prophylactic anti-oxidant. Time to development varies between organs and rapid development is more associated with suboptimal organs. Its insidious development can lead to organ injury resulting in NMP failure. *N*-acetylcysteine is an anti-oxidant used commonly in liver injury and works effectively through the synthesis and replenishment of hepatocyte glutathione stores. *N*-acetylcysteine is a potent, yet safe, and cheap anti-oxidant which when added as a continuous supplement during extended NMP efficiently prevents accumulation of methaemoglobin.

### Supplementary Information

Supplementary Table 1.

## Data Availability

The datasets generated during the current study are available from the corresponding author on reasonable request.

## Abbreviations

CIT: Cold ischaemia time
DBD: Donation after brainstem death
DCD: Donation after circulatory death
ECD: Extended criteria donor
Hb: Haemoglobin
MetHb: Methaemoglobin
NAC: *N*-Acetylcysteine
NHSBT: National Health Service Blood and Transplant
NMP: Normothermic Machine Perfusion
