# Supplementary material for: N-acetylcysteine: a novel approach to methaemoglobinaemia in normothermic liver machine perfusion
Source: Sci Rep. 2023 Nov 3;13:19022. doi: 10.1038/s41598-023-45206-z (PMC10624848; doi:10.1038/s41598-023-45206-z)
Supplement: Supplementary file 1 — Supplementary Table 1. [file 41598_2023_45206_MOESM1_ESM.docx]

|  | 1 | 2 | 3 | 4 | 5 | 6 | 7 | 8 | 9 |
| --- | --- | --- | --- | --- | --- | --- | --- | --- | --- |
| Device used | Liver Assist | | | | | | | | |
| Oxygen Carrier | 3 units human packed red cells | | 4 units human packed red cells | | | 5 units human packed red cells | | | |
| Perfusion Constituents | 10% Calcium gluconate  8.4% Sodium bicarbonate  Heparin  Gentamicin  Vancomycin  Human Albumin Solution | | | 10% Calcium gluconate  8.4% Sodium bicarbonate  Heparin  Piperacillin with Tazobactam  Human Albumin Solution  N-acetylcysteine | 10% Calcium gluconate  8.4% Sodium bicarbonate  Heparin  Piperacillin with Tazobactam  Human Albumin Solution  Methylprednisolone  N-acetylcysteine | | | | |
| Infusions | Epoprostenol  Sodium Taurocholic Acid  Aminoplasmal (w/ cemevit + phytomenadione)  Heparin  Insulin  Glucagon  50% dextrose | Epoprostenol  Sodium Taurocholic Acid  Aminoplasmal (w/ cemevit + phytomenadione)  Heparin  Insulin  Glucagon  50% dextrose  Noradrenaline  Sodium Lactate Solution | Epoprostenol  Sodium Taurocholic Acid  Aminoplasmal (w/ cemevit +  phytomenadione)  Heparin  Insulin  Glucagon  50% dextrose  Noradrenaline  Argipressin  Sodium Lactate Solution | Epoprostenol  Sodium Taurocholic Acid  Aminoplasmal (w/ cemevit + phytomenadione)  Heparin  Insulin  Glucagon  50% dextrose  Noradrenaline  Argipressin  Sodium Lactate Solution  Piperacillin with Tazobactam  N-acetylcysteine | Epoprostenol  Sodium Taurocholic Acid  Aminoplasmal (w/ cemevit +  phytomenadione)  Heparin  Insulin  50% dextrose  Sodium Lactate Solution  Piperacillin with Tazobactam  Methylprednisolone  N-acetylcysteine | | | | |
| CVVH | No | | Yes | | | | | | |
| N-acetylcysteine used | No | | | Yes | | | | | |
| Total Perfusion Time | 102 hours | 100 hours | 87 hours | 90 hours | 121 hours | 156 hours | 184 hours | 168 hours | 168 hours |

**Supplementary Table 1:**

Table highlighting the perfusion protocol differences between Livers 1 – 9.
